# Sun-Exposed versus Sun-Protected Cutaneous Basal Cell Carcinoma: Clinico-Pathological Profile and p16 Immunostaining

**DOI:** 10.3390/diagnostics13071271

**Published:** 2023-03-28

**Authors:** Abdulkarim Hasan, Ahmad M. Kandil, Hasan S. Al-Ghamdi, Mohammad A. Alghamdi, Mohamed Nasr, Suhaib Alsayed Naeem, Wagih M. Abd-Elhay, Osama Khalil E. Mohamed, Hany Sabry A. Ibrahim, Eman Mohamed Ahmed, Ahmed Elsayed M. Abdrabo, Shimaa Abdelraouf Elgohary

**Affiliations:** 1 Pathology Department, Faculty of Medicine, Al-Azhar University, Cairo 11884, Egypt; 2 Internal Medicine Department, Division of Dermatology, Faculty of Medicine, Albaha University, Albaha 65799, Saudi Arabia; 3 Histology Department, Faculty of Medicine, Al-Azhar University, Cairo 11884, Egypt; 4 Dermatology, Venerology and Andrology Department, Faculty of Medicine, Al-Azhar University, Cairo 11884, Egypt; 5 Dermatology, Venerology and Andrology Department, International Islamic Center of Population Studies and Research, Al-Azhar University, Cairo 11651, Egypt; 6 Pathology Department, Faculty of Medicine for Girls, Al-Azhar University, Cairo 11884, Egypt; 7 Community and Industrial Medicine Department, Faculty of Medicine, Al-Azhar University, Cairo 11884, Egypt; 8 Pathology Department, Faculty of Medicine, Ain Shams University, Cairo 11517, Egypt

**Keywords:** basal cell carcinoma, immunohistochemistry, non-melanomcytic, p16, skin cancer

## Abstract

Introduction: Although widespread, BCC is still relatively poorly understood in regards to pathogenesis and prognosis, particularly the lesions formed on anatomical sites away from sun exposure. With the aim of deepening our understanding of the pathogenesis and clinico-pathological correlations of BCCs, we conducted this study. Methods: Tissue blocks and data of 52 Egyptian patients diagnosed with BCC were retrieved for clinical information and inclusion criteria, then re-examined histologically; p16 immunostaining was carried out and evaluated for analysis and comparison between the two groups, i.e., sun-exposed and sun-protected. Results: Sex, age, clinical suspicion, tumor size, recurrence status, and histologic variants did not show a significant difference between the sun-protected and sun-exposed groups; however, the mean ages recorded were 67.2 vs. 62.7 for the sun-protected and sun-exposed groups, respectively. A total of 52% of BCCs were positive for p16. The sun-protected lesions showed p16 positivity in 61% of cases, whereas 49% of the sun-exposed lesions were positive with no significant difference. There was a significant difference in p16 expression between the recurrent and non-recurrent lesions. Conclusions: A significant difference was seen in the case of cancer recurrence, where all the recurrent BCCs in this study demonstrated negative p16 immunostaining of the primary lesions; however, the positively stained cases in total were 52% of BCCs. The mean patient age of the sun-protected group was much higher than in previous peer studies. We assume that the biological, prognostic, and clinical aspects of p16 protein expression in BCCs are still far from being clearly understood. Further studies are highly recommended, with more focus on its role in the pathogenesis and the prognostic factors.

## 1. Introduction

Basal cell carcinoma (BCC) is an immunogenic neoplasm for which pathogenesis associates strongly with environmental and genetic factors in addition to several other patient-dependent factors [1].

Skin cancers, including BCC, in their early stages, will be mediated through a wide range of effects. The gene-related expression changes indicate their association with ultra-violet (UV) radiation, which is the most well-known environmental carcinogen of the skin [2]. Therefore, sun exposure is the most known environmental cause of cutaneous BCC of importance, and the risk of this cancer appears to depend on the nature of exposure. A population-based, case-control study conducted in Canada revealed an increased risk of cutaneous BCC with recreational sun exposure in the childhood and adolescent life periods, suggesting that these periods may be relatively critical for establishing the risk of BCC [3].

Although exposure to solar ultraviolet radiation is considered the main cause of the majority of BCCs, epidemiologic studies suggest that the quantitative increase in the cancer risk is at best modest, with self-reported high, compared with low, sun exposure typically associated with less than a doubling of the risk of BCCs. This is unlike squamous cell carcinoma, which is directly associated with a cumulation of sun exposure [4]. The association between ultraviolet rays and BCC is not straightforward, as the association between chronic sunlight exposure and BCC is modest, and markers of cutaneous sun damage are associated (only) moderately with an increased risk of BCC. Approximately one-quarter of the BCCs worldwide occur on anatomic sites that are not habitually exposed to sunlight, such as the trunk and genitalia [5]. Therefore, the risk factors for BCC may vary according to the anatomic site location at which they arise, and other factors should be studied in detail.

The p16 gene is supposed to be involved in the pathogenesis of cutaneous BCCs in view of increased p16 mRNA and also the expressed protein within tumor cells [6,7]. p16, a tumor suppressor, is a biomarker for transforming HPV infections and is validated as an accurate surrogate marker for HPV status determination in various types of tumors [8,9]. The p16/Rb/E2F regulatory pathway participates in cell cycle arrest and is inactivated in most human cancers, whereas p16INK4a is linked with CDK4 and CDK6 in competition with cyclin D1, which then prevents phosphorylation of the tumor-suppressor protein retinoblastoma (Rb), which contributes to form the pRbeE2F growth inhibitory complex and plays an important role in tumor pathogenesis [10].

Human papilloma viruses (HPVs) are highly prevalent in human populations and are classified as alpha (α), beta (β), gamma (γ), mu (μ), and nu (ν) genera [11]. Based on the frequency of HPVs, they can be divided into (1) cutaneous types, commonly seen in benign skin warts; (2) mucosal types, detected in genital condylomas and ano-genital cancers; and (3) epideromodysplasia verruciformis types, now indicated as beta-HPVs [12,13].

Some of the cutaneous beta-HPVs have been suggested as co-factors in the development of non-melanoma skin cancer (NMSC), including the most common form, BCC [14,15,16]. HPVs are increasingly recognized as important human carcinogens, but their role in the etiopathogenesis of BCC is unclear, as the majority of studies on HPV’s role in the pathogenesis of NMSCs have focused on squamous cell carcinoma and there is a limited number of reports and studies regarding HPV infection in BCCs [13,17].

Bartoš in 2020 [17] questioned whether p16 protein production in tumor cells depends on the topographic distribution of lesions and if it may be influenced by solar exposure. Some authors [17,18] have found that p16 overexpression is associated with skin cancer arising in the sun-exposed area, suggesting a possible induction of p16 production by permanent UV radiation. On the other hand, some researchers [19] have not found such associations in the studied BCCs. Furthermore, Villada et al. [20] recorded ten p16-negative BCCs situated on the head and neck in contrast to other anatomical sites studied for p16. With the aim of establishing a deeper understanding of the pathogenesis and prognosis of BCC, we compared sun-exposed with sun-protected lesions in regard to p16 expression in addition to the correlation with some demographic and clinico-pathological features of the included patients, including age, sex, tumor size, state of recurrence, and the histological variant.

## 2. Methods

### 2.1. Patients

This retrospective cross-sectional study included 52 BCCs from 52 patients (21 males and 31 females). A total of 29 tumors were from sun-exposed anatomical sites, and 24 were from sun-protected sites, which were selected randomly from histopathologically examined lesions after surgical excision at our university hospital in Cairo during the period from 2017 to 2020 and after providing research ethics committee approval. Inclusion criteria included all patients of all ages who came to the dermatology or surgery departments with lesions suspected of skin cancer and which were confirmed as BCC according to histological/pathological examination; the tissue blocks were preserved in the laboratory. Patients who underwent incisional biopsies or incomplete excision of the lesion, patients lacking follow-up data, and those with the inability to provide the related clinical or pathological data were excluded from the study.

### 2.2. Clinical Information

The clinical data were retrieved from the histopathology report or medical files of the included patient record, including patient age, sex, clinical examination and suspicion, and history of sun exposure to the anatomical area where the BCC formed. The sun-exposed areas include the face, scalp, hands, feet, and the forearm in males; female patients with forearm lesions were excluded from this study as we were not sure exactly if the patient usually covered this part of the body or not. The size of the tumor and least free margin distance, in addition to the recurrence status of the lesion for three years, were also recorded.

### 2.3. Histopathological Staining and Examination

Histopathology reports were carefully retrieved, and the histological variant for each case was recorded after re-examination of the slides under a light microscope. If approved and agreed upon by at least two histopathologists, the tissue blocks were re-cut to prepare for immunohistochemical study.

Tissue blocks were collected according to the inclusion criteria from the medical files, pathology reports, and related clinical data, in addition to the availability of the tissue blocks in our institution after the ethics committee’s approval. Extra sections were cut by the histotechnician using the microtome machine. The slides were air-dried, fixed with ethanol, and rehydrated in descending alcoholic solutions (in dosages of 100%, 90%, 75%, and 50%). The slides were rinsed in distilled water for five minutes. Then, they were waterlogged for three to four minutes in filtered hematoxylin (H) stain and rinsed twice with distilled water. After that, the slides were waterlogged for five to seven seconds in filtered eosin (E) stain, followed by rinsing with distilled water. The following step was soaking the slides in xylene solution, mounting them with Canada balsam material, and allowing them to dry with the coverslips in place using a specific machine for covering. On each slide, careful histologic examination was performed by at least two blinded histopathologists who gave their microscopic description, diagnosis, variant detection, and marginal status with measurement using the microscope’s vernier scale. Random representative microscopic fields were photographed at different magnification power degrees. This was accomplished with digital cameras (Canon, Japan) attached to the provided light microscope.

### 2.4. p16^INK4a^ Immunohistochemical Study

The p16^INK4a^ immunostaining was carried out on tissue sections of 5 μm thickness from formalin-fixed and paraffin-embedded blocks in a two-step process involving the binding of primary antibodies to the antigens of interest, followed by the detection of bound antibodies by the chromogen according to the manufacturer’s instructions. Mouse monoclonal antibodies for HPV (Anti-Papillomavirus Type 16 (known as HPV-16), clone: Cam vir-1, ready-to-use kits, BioGenex, CA94538) and mouse monoclonal antibodies to P16 (Anti-P16/INK4A, clone: G175-405, ready-to-use catalog No. AM540-5M, BioGenex, CA94538) were used in the laboratory as primary antibodies. The first antibodies stain the nucleus in positive cells. The second antibodies stain the nucleus +/− cytoplasms in positive cells. The positive controls for this marker were known cases of cervical carcinomas in biopsies for P16 and known cases of SCC of the skin.

### 2.5. Scoring of the p16 Immunostaining

Nuclear staining, with or without cytoplasmic reactivity of p16^INK4a^, was considered positive, and a percentage of the positive nuclei was calculated. Cases were then divided into 3 categories according to the number of p16-positive cells: negative = less than 1% positive nuclei, weak positive = 1–30% positive nuclei, moderately positive = 33–70%, and strong = 70% or more positive nuclei. Similar scoring was previously employed for p16INK4a expression in nonmelanoma skin cancer [21]. Staining intensity was not assessed to avoid subjective interpretation.

### 2.6. Ethical Approval

This study was approved by Research Ethics Committee at Al-Azhar Faculty of Medicine, under ID number: His_395Med.Research_00000105; the study was conducted in accordance with the Declaration of Helsinki (2013) and the national ethical review of biomedical research. Written informed consent was waived due to the retrospective nature of this study design, and the data were maintained with high confidentiality to ensure the participants’ privacy.

### 2.7. Statistical Analysis

Data are expressed as numbers and percentages (qualitative data) or as means and standard deviations (quantitative data). Comparisons were carried out using, for qualitative data, the chi-square test when its assumptions were met, or alternatively Fisher’s exact test or the Monte-Carlo method. Quantitative data comparison was performed using an independent samples *t*-test when assumptions were met; alternatively, the Mann–Whitney U test was used. IBM^®^ SPSS^®^ software was used for conducting the required analyses. All statistical tests were performed at the 0.05 level of significance.

## 3. Results

### 3.1. Demographic Data Comparison

The differences in the distribution of sex and age between the sun-protected and sun-exposed groups were not significant. Age had a mean ± SD of 67.2 ± 10.25 vs. 62.7 ± 14.35 for the sun-protected and sun-exposed groups, respectively (*p* = 0.216). Males made up 43.5% of the sun-protected group compared to 37.9% of the sun-exposed group (*p* = 0.69).

### 3.2. Clinical Data Comparison

**Figure 1:**
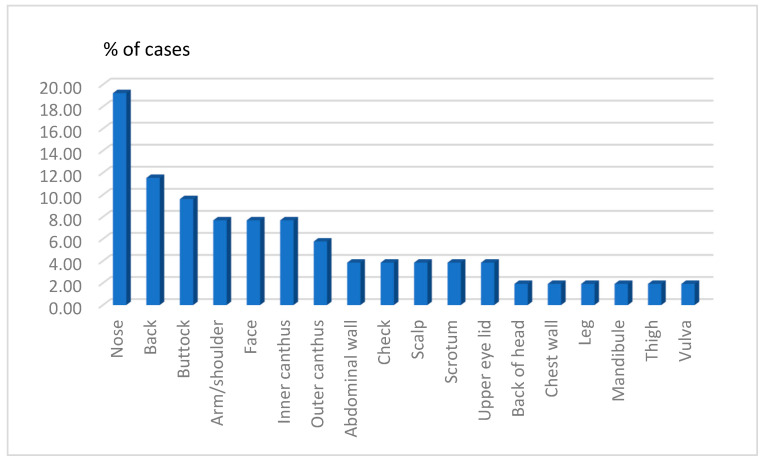
A bar chart demonstrating the anatomical distribution of the total studied cases.

The topographic distribution of the studied cases is displayed in the bar chart (Figure 1). The participant groups and anatomical site distribution of the participants were categorized into two groups. The first were those who had BCC on sun-exposed areas according to the clinical examination and patient’s medical history after the exclusion of patients with an uncertain history of exposure, including 29 lesions in 29 patients: 10 lesions were seen on the nose, either on the front or both sides of the nose; 7 lesions on the eye canthus (4 inner and 3 outer canthus); 4 lesions on the face without specification of a particular site; 3 on the scalp (2 lesions on the scalp and 1 written on the request form as a back-of-head lesion); 2 tumors on the upper eyelids; 2 lesions on the cheek areas; and 1 lesion on the skin overlying the mandible in a 14-year-old boy, who was the youngest participant in this study.

The second were those who had BCC on sun-protected areas according to the clinical examination and retrieval of the patient’s medical history, as well as clinical photos taken when applicable, including 23 patients with 23 lesions as follows: 6 lesions on the back (4 from women and 2 from men after assurance of non-exposure to sunlight); 5 seen on the buttocks or gluteal region; 4 lesions on the upper arm and/or shoulder in female patients; 2 tumors on the abdominal wall skin; 2 lesions on the scrotal area in two patients of 70 and 71 years old; 1 lesion on the vulva in a 70-year-old woman; 1 lesion on the right thigh skin in a 49-year-old man; 1 lesion on the chest wall skin in an 82-year-old man; and 1 tumor on the leg skin in a 67-year-old man.

No significant difference was seen between sun-exposed and sun-protected lesions in regards to tumor size, recurrence, or the clinically appearing basal carcinomatous lesions; however, 55.2% of the sun-exposed lesions were clinically suspected BCCs, whereas only 35% of the lesions on sun-protected areas were diagnosed clinically as BCCs (Table 1). P16 status showed a significant difference between recurrent and non-recurrent lesions across all cases (Table 2).

### 3.3. Histological Features

**Figure 2:**
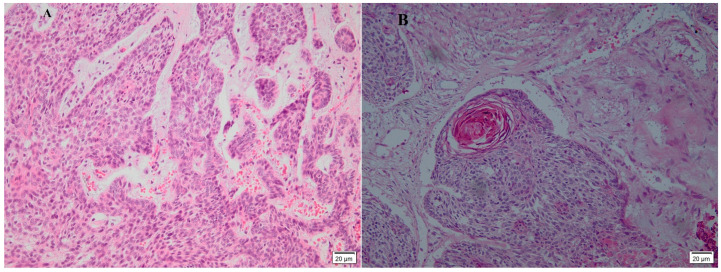
A histopathology picture of basal cell carcinoma showing dermal basaloid malignant nodular masses in the dermis with peripheral palisading and cleft seen area (H&E, 200×), (**A**): Myxoid stroma appears. (**B**) The cleft is obvious around the tumor.

The majority of the total cases (67.3%) were nodular variants, and the remaining one-third of the total cases were other variants, as shown in Table 1. The nodular variant represented two-thirds of the total cases, showing relatively circumscribed masses exhibiting large basaloid lobules with peripheral nuclear palisades and cleft formation between tumor lobules and stroma. Lobules may be solid or may show central cyst formation with excessive mucin production. Most cases showed mild pleomorphism with variable mitotic activity and apoptosis (Figure 2).

### 3.4. P16 Immunostaining

**Figure 3:**
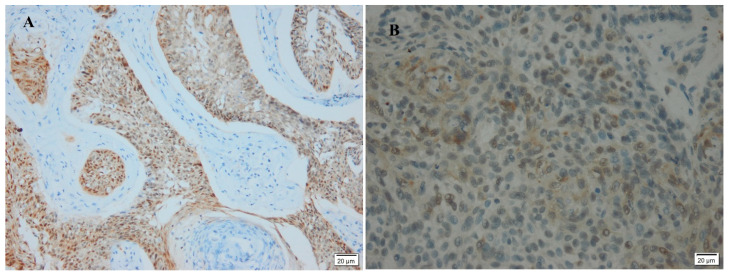
Immunohistochemical staining of p16 marker in a case of BCC showing: (**A**) strong positivity (200×); (**B**) moderate positivity in (400×).

**Figure 4:**
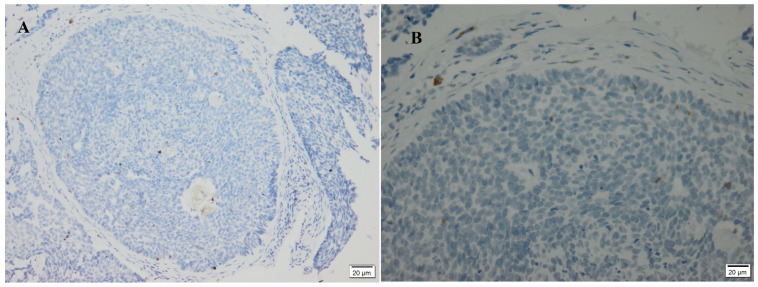
Negative immunostaining of p16 in two cases of BCC. (**A**) A case from a sun-exposed area (200×); (**B**) a case from a sun-protected area (400×).

A total of 24/52 (48%) BCCs in this study were negative for p16, and 26/52 (52%) were positive (Figure 3 and Figure 4). The sun-protected lesions showed p16 positivity in 61% of cases, whereas 49% of the sun-exposed lesions were positively stained, with no significant difference (Table 1). There was no significant difference between box age groups (>50 years old and <50), both genders (male and female), tumor size (>20 mm and <20 mm), or the histological variant (Table 2). A significant difference in the p16 immunostaining appeared in the case of recurrence.

## 4. Discussion

Basal cell carcinoma is one of the most common skin cancers diagnosed worldwide, affecting more than 3 million people in the United States each year, and it commonly occurs in sun-exposed anatomical skin sites, especially the head and neck region [22]. Although BCCs are usually indolent, non-aggressive tumors that can only invade their surrounding tissues locally, some lesions are locally destructively aggressive and can occasionally recur or metastasize with a metastatic rate of 0.1% to the lymph nodes [23].

Basal cell carcinoma of the skin can be considered low risk when it is located on the trunk or the extremities and measures less than 1 cm in diameter if the patient is immunocompetent [24]. In this study, 11 patients had a tumor with a maximum diameter of more than 20 mm; this is quite a large size but may reflect some sort of neglect or late diagnosis of such skin lesions in developing countries. Histological subtypes of low-risk cancers include superficial and nodular variants of BCCs; however, the infiltrating, micronodular, and morphoeic variants are counted as high-risk variants. Low-risk lesions lack perineural invasion, while high-risk lesions are larger in size and mostly affect the trunk, extremities, and middle of the face. High-risk BCC tends to be recurrent [25,26].

As the population ages worldwide, the incidence of BCC continues to increase; the reported median age of patients diagnosed with BCC is 67 years, and the incidence increases with age [27]. Some studies suggest that male gender at age > 60 years poses a relatively increased risk of cutaneous BCC recurrence [28]. However, more recent studies suggest a lack of significance for age and gender in this important prognostic sign (recurrence) [29]. Although ultraviolet (UV) radiation is the major risk factor for BCCs, their anatomical distributions differ, as BCC occurs primarily on sites commonly exposed to sunlight, especially the face and the upper and lower limbs, but is also seen in areas less frequently exposed to the sunlight [30,31]. Different clinico-histologic subtypes and different anatomic sites of BCC may display distinct pathogenesis and different characteristics. A study by Pyne et al. found that sun exposure was associated with deeper BCC invasion [32].

However, scant information and studies exist on potential differences in the etiological factors for BCCs according to anatomic location [33].

Many researchers have studied the correlation between skin tumor pathogenesis and HPV infection, but so far HPV variants with a putative increased potential for malignancy have been observed only in squamous cell carcinoma (SCC) in most cases, in a few patients of the rare autosomal inherited disease epidermodysplasia verruciformis, and in some cases of non-melanoma skin cancer in immunosuppressed transplant patients [19,34]. The relationship between HPV infection and BCC is still not consistent, with the causative role and the data on this topic still limited; however, an association between beta-HPV and the expression of p16^INK4a^ and Akt has been found, as they are involved in cell cycle deregulation [19].

About half of the BCCs in this study showed p16 positivity; all were from Egypt, with a mean age of 62.7 and 67.2 in sun-exposed and sun-protected cases, respectively, and a female predominance.

A peer study from France recorded a similar percentage (50%) of p16 expression in cutaneous BCCs [18]; however, a study from Iran recorded a much higher percentage (79%) of positive staining BCC cases [2]. A greater difference was identified in a study carried out by Villada et al. [20], who did not report any p16-positive BCC in their study in 2018.

The slight increase in the mean age of the sun-protected group was inconsistent with previous studies, which recorded a lower mean age at occurrence of BCC observed in non-sun-exposed anatomical areas [35,36,37]. Those studies suggested that the non-melanoma skin cancers of sun-protected sites may occur in individuals with decreased capacity for DNA repair; however, we believe that the exact pathogenesis should be further studied. Regarding p16, we found no significant association between its over-expression and the anatomical site or sun exposure.

Our results are more consistent with those of Svensson et al. and Eshkoor et al., who did not find evidence of such associations [8,19,38]. On the contrary, Conscience et al. [18] showed a p16 over-expression in BCC located in sun-exposed areas, reflecting a significant association. No association could be detected between the histological variant and p16 expression, which is consistent with previous studies [18,19]. The histologic type was also not associated with sun exposure, as different variants were seen in both groups with no significant differences.

The nodular variant was the predominant variant in our study, which is consistent with most of the literature revealing that the nodular subtype is the most common variation, accounting for 50 to 79% of all BCCs [36,39]. In one study, 90% of nodular BCCs were seen on the head and neck region [36]. Clinically, the tumor may be enlarged and show crusting over a central depression; bleeding with minor trauma is not uncommon and the lesion may ulcerate (called a rodent ulcer), but a rolled border remains, serving as a clue to the clinical diagnosis. Regarding the clinical diagnosis of BCCs, we recorded around 50% of the total cases as correctly clinically diagnosed as basal cell carcinoma. This study included four patients who experienced a rare condition of BCC recurrence; all the primary lesions revealed negative p16 immunostaining. Previous studies have shown no definite correlation between subtype and recurrence of BCC, and the histopathological criteria for prognosis are still limited, with no sufficient data on the role of p16 in recurrence [40,41].

The loss of nuclear p16 expression in some cases of melanomas is associated with increased Ki-67 expression (tumor cell proliferation) and vascular invasion, which independently predict decreased patient survival, according to Straume et al. [40]. We recommend further studies on the prognostic role of p16 expression in BCCs.

The limitations of the current study include a lack of determination of some related clinical history, such as sun exposure periods; a lack of detection of HPV DNA using the related primers or in situ hybridization (ISH) due to financial issues; the absence of genodermatosis in the included boy in this study; and, lastly, an inability to assess the normal skin p16 status of the included cases because the nature of this study depended on studying only the available tissue blocks in the pathology and dermatology departments.

## 5. Conclusions

Our data demonstrate that there is no significant difference between sun-exposed and sun-protected groups in regards to the age and gender of patients; however, the mean age of sun-protected group patients was 67, which is much higher than the recorded mean in peer studies in other geographic areas. No association could be detected between p16 expression and topographic distribution, tumor size, or the histological variant of cutaneous BCC. However, nearly 50% of the total BCCs showed positive p16 immunostaining. The only significant difference was seen in cases of recurrence, where all the recurrent lesions in this study demonstrated negative p16 immunostaining of the primary lesions. We assume that the biological, prognostic, and clinical aspects of p16 protein expression in BCCs are still far from being clearly understood. Further studies are highly recommended, with more focus on the pathogenesis and the prognostic role of similar markers.

## Figures and Tables

**Table 1:** Demographic and clinic-pathological features.

		Sun-Protected (*n* = 23)	Sun-Exposed (*n* = 29)	*p*-Value
**Demographic data**				
Age, mean (SD)		67.2 (10.25)	62.7 (14.35)	0.216
Sex, no. (%)	Female	13 (56.5)	18 (62)	0.69
	Male	10 (43.5)	11 (37.9)	
**Clinical data**				
Clinical diagnosis, no. (%)	BCC	8 (34.8)	16 (55.2)	0.17 ^f^
	Other than BCC	15 (65.2)	13 (44.8)	
Tumor size in mm, mean (SD)		13.1 (8.8)	13.7 (9.6)	0.81
Recurrent state, no. (%)	Recurrent	1 (4.3)	3 (10.3)	0.06 ^f^
	Not recurrent	22 (95.7)	26 (89.7)	
**Histopathological examination**				
Histological, variant, no. (%)	BCC nodular	18 (78.2)	23 (79.3)	0.31 ^m^
	BCC Superficial	4 (17.4)	5 (17.3)	
	BCC adenoid	0 (0)	1 (3.4)	
	BCC keratotic	1 (4.3)	0 (0)	
**P16 status**				
P16 immunostaining, no. (%)	Negative	9 (39.1)	15 (51.7)	0.37
	Positive	14 (60.9)	14 (48.3)	
P16 expression, categories, no. (%)	Strong positive	1 (4.3)	4 (13.8)	0.27 ^m^
	Moderate	12 (52.2)	9 (31.1)	
	Weak	1 (4.3)	1 (3.4)	
	None	9 (39.2)	15 (51.7)	

The above comparisons were conducted using independent samples *t*-test for ages, or chi-square test, Fisher’s exact test (^f^), or Monte-Carlo method (^m^), as appropriate for qualitative variables. Significant *p*-value at 0.05 level.

**Table 2:** P16 status in correlation to other clinico-pathological data.

		P16+	P16−	*p*-Value
Age	<50 years old	3	2	0.1 ^f^
	≥50 years old	25	22	
	Total	28	24	52
Sex	Female	17	14	0.86
	Male	11	10	
	Total	28	24	52
Recurrence	Yes	0	4	0.039 * ^f^
	No	28	20	
	Total	28	24	52
Tumor size	<20 mm	21	20	0.46 ^m^
	≥20 mm	7	4	
	Total	28	24	52
Histological variant	Nodular	22	13	0.061
	Non-nodular	6	11	
	Total	28	24	52

The above comparisons were conducted using chi-square test, Fisher’s exact test (^f^), or Monte-Carlo method (^m^) as appropriate for qualitative variables. *: significant *p*-value at 0.05 level.

## Data Availability

The data that support the findings of this study are available from the corresponding author upon reasonable request.
